# Involving Patients in Research? Responsible Research and Innovation in Small- and Medium-Sized European Health Care Enterprises

**DOI:** 10.1017/S0963180118000488

**Published:** 2019-01

**Authors:** KALYPSO IORDANOU

**Keywords:** RRI, health care, reasoning, responsible research

## Abstract

Health research is generally undertaken to resolve existing health problems or enhance existing solutions. Research ethics committees have been the main governance tool for research for more than half a century. Their role is to ensure that research is undertaken ethically. To close the increasing gap between science and society, other governance tools are required. The European Commission recommends and actively promotes the policy of responsible research and innovation (RRI). In addition to sound research ethics, a key feature of RRI is the involvement of different societal stakeholders throughout the research process.

But how accepted is the involvement of societal stakeholders in the research of small- and medium-sized enterprises (SMEs) in the health care sector? This question is examined based on 18 in-depth interviews with private health care industry representatives from across Europe in companies focusing on developing medical device technology. Findings suggest that SMEs are reluctant to undertake research involving patients, especially in the early stages of the research and innovation process. For some SMEs this is due to concerns about the dangers of raising expectations they cannot meet, while for others the main concerns are increasing costs and producing less competitive products. Implications of the research findings are discussed.

## Introduction

An increasing gap has been observed between research and the science and society interface. The reasons for this gap are manifold, ranging from an uneasiness about emerging technologies^1^ to a mismatch of research with societal needs. The U.S. National Academy of Sciences recently attributed this phenomenon to the hypercompetitive culture of the academic world, especially in the field of the biomedical sciences.^2^ Hypercompetitiveness in academia drives researchers to invest their efforts in topics that promise to lead to results that will be publishable in elite, high-impact journals, exaggerating the presentation of significant findings and omitting the presentation of nonsignificant results.^3^

Poor research practices have detrimental consequences on the quality of research outputs, and can result in publications whose results cannot be replicated.^4^ Low-quality research has limited benefits for society and constitutes a waste of public money, given that a significant number of research studies are supported by public funds. For example, there is evidence that a high proportion of the quarter of a trillion U.S. dollars that are spent every year globally on research in health care are wasted due to bad practices in research.^5^ This includes poor choice of research questions that do not build on existing research findings and biased data reporting.^6^

A series of recommendations were published in the Lancet series in 2014 about health care research,^7^ addressed to five main stakeholders: funders, regulators, journals, academic institutions, and researchers themselves. However, another stakeholder has received much less attention in policy recommendations—the private health care industry. This is a wide sector that encompasses everything from manufacturing medical equipment and pharmaceuticals to operating health care facilities. A considerable amount of this research is conducted outside of traditional university laboratories; in fact, the business enterprise sector is the largest performing sector in research and development, accounting for 64% of total research and development expenditure in the European Union (EUR 191.2 billion), which is three times more than the higher education sector spends (EUR 69.4 billion).^8^

The rapid growth of start-ups, which combine research, innovation, and enterprise, and their increasing rate of development, suggests that we need to take research conducted by industry into consideration in any attempt to address the gap between research and society; any efforts to bridge the gap between research and society that exclude industry are condemned to fail. Therefore, there is an urgent need to enrich our understanding of how research is conducted in the private sector and particularly the alignment of industry research with the recommendations of the European Commission for early and continued stakeholder involvement.

## Responsible Research and Innovation

The European Union has introduced the nuanced term responsible research and innovation (RRI) in an effort to bring research closer to society. RRI is an approach that aims to foster the design of inclusive and sustainable research and innovation. RRI recommends collaboration between different societal actors—including researchers, citizens, policy makers, and business—throughout the process of research and innovation in order to better align both the process and its outcomes with the values, needs, and expectations of society.^9^ RRI can be implemented through public engagement, open access, taking gender and ethics into consideration, and formal and informal science education.^9^

A key factor of the successful implementation of RRI in industry is decision-makers’ reasoning and attitudes towards RRI. There is a close connection between individuals’ beliefs and their behavior.^10-12^ If decision-makers do not see the value of RRI, they will not promote its application. Chatfield, Borsella, Mantovani, Porcari, and Stahl^13^ examined the attitudes of individuals working in the information and communication technology (ICT) industry. They found that the business risk of a gap between science and society is underestimated in the ICT industry. They recommend that better awareness of the full range of ethical and societal risks involved in research and innovation amongst industry leaders could increase RRI uptake. Yet our understanding of how RRI is perceived in industry in areas other than ICT is still limited.

## The Engagement of Stakeholders in the Process of Research and Innovation in the Health Care Sector

According to the World Health Organization,^14^ in order to be able to reach the goal of offering better health for all, it is imperative to increase stakeholder participation and bring people into the foreground by organizing health services around people’s needs and expectations. The role of patients in health care research has received increasing attention and it is considered to be an imperative for promoting medical innovation and improving the quality of health care.^15-17^ Yet our knowledge of the role of patients in health care research in many areas of the private sector is limited. The present study aimed to shed light on the application of RRI in industry, focusing on the process of research and innovation for medical device technologies in the health care sector. The study examines the reasoning of key industry representatives from across Europe regarding RRI. Drawing upon empirical data from 18 in-depth interviews, the study explores the level of awareness of RRI and the extent to which patients are involved in the process of research and innovation. The findings of the study have the potential to inform our understanding of the gap between research and society.

## Methods

### Participants

Participants were 18 key industry representatives across Europe whose focus is developing medical device technologies in the health care sector. Seven of the participants were the founders of companies, and 11 held high-level managerial positions (chief executive officers or managing directors) in SMEs. Five participants were from the U.K., 5 from Austria, 4 from Spain, 3 from Cyprus, and 1 from Slovenia (see **Table 1**).

**Table 1.** Overview of Participants

Country	Position held
Spain	CEO
Cyprus	Founder
Spain	CEO
Cyprus	CEO/Founder
Cyprus	CEO/Founder
Spain	Director
Austria	CEO
Spain	IT Manager and Managing director
Austria	Founder
Austria	Founder
Austria	Founder
Austria	Founder
U.K.	Chairman
U.K.	Chief executive officer of U.K. subsidiary
U.K.	National sales manager
Slovenia	Managing director
U.K.	Managing director
U.K.	Managing director

Purposeful sampling was conducted to identify prospective interviewees in different countries and ensure that the participants had the relevant experience and were able to provide information regarding the decision-making processes of their companies. It was vital that all participants, including the founders, held current managerial positions in the SMEs and were actively involved in research and innovation in their companies.

### Instruments, Data Collection, and Analysis

An interview protocol was developed to ensure consistency in the data collection. The interview protocol was based on the protocol that was developed by Chatfield and Iatridis et al.^18^ It was revised and finalized after receiving feedback from three experienced researchers in RRI.

Eighteen in-depth interviews were conducted. The interviews were carried out by three researchers located in the U.K., Austria, and Cyprus. The task of the interviewers was one of probing for further details or asking for clarification when necessary; the interviews proceeded as a conversation rather than a question-and-answer session.

Analysis of the transcripts was undertaken centrally, led by the author, to ensure consistency. A stepped process of thematic coding was utilized. An inductive approach was used. The first stage of open coding was followed by a further stage of thematic coding during which emerging themes were compared and contrasted and gradually refined. Two other researchers, who were also involved in the data collection, reviewed the transcripts of the interviews and the themes that emerged and provided feedback. After discussion among the three researchers, the themes were finalized.

## Findings

### Involvement of Different Stakeholders in the Research and Innovation Process

When participants were asked whether they take into consideration a wide range of stakeholders in the research and innovation process in their organization, 4 (22%) reported that they involved universities and other companies, 3 (17%) reported that they involved experts, hospitals, doctors or other practitioners (e.g., nurses, occupational therapists, social workers), and 2 (11%) reported that they involved the local authority.

Some interviewees underlined that there are conflicting interests among stakeholders, which make their inclusion in research and innovation in their company challenging. For example, one participant said the following:

There are some stakeholders that . . . have invested in the company, so that research has to be oriented to create value. The government is always involved because there are always grants or loans or subloans that can help the company to develop a specific part of that research.

Notably, none of the interviewees reported engagement of patients. Some participants reported collaboration with doctors or hospital administrators, but not with patients themselves.

### Inclusion of Patients in the Development Processes

Interviewees were asked whether consideration is paid to their target or end users in research and innovation activities. Most of the responders mentioned that feedback is requested in an unsystematic or informal way, while some of them noted that little consideration is paid to users in research and innovation activities in their companies.

Five (28%) reported that they asked for and received feedback from the end users about their satisfaction with the products. Some of the interviewees did not mention patients at all in their responses, answering this question by explaining that they collaborate with universities and medical experts in research and innovation activities in their companies. One interviewee elaborated that patients were not taken into consideration in decision-making in research and innovation activities in industry:

The hospital is the only decision maker at the end of the day. The physician can make pressure in case the product is so good that he desperately wants it. Sorry for saying this but the interest of the patients is not really there and the patients are a very small lobby in this situation. Maybe the patients have a strong lobby for public things but technology-wise and medtech-wise, . . . the physician decides a little bit but mainly the hospital decides if this technology is used or not.

Notably, participants who mentioned that patients were consulted in their companies reported that this involvement took place not during the development of the products, but only later, for improving the products after they had been developed. One of the interviewees explained why patients were involved only at the final stages of product development—this decision was justified by explaining that earlier involvement of patients can be detrimental for the development of the product:

R: If you’re thinking more about patients or patients associations, those are really far away from what the company needs at that process and even can be . . . not helpful, even can . . . damage the process itself.

Interviewer: Why damaging?

R: Well because of expectations. When you’re talking to patients or associations of patients, they want solutions on short-, medium-term. When you’re investing in early stage you are thinking about 20 years to have the solution on the market. So that disappointment in your research could damage the image of the research because if you don’t deliver, they can think that you are not going in properly or say that you are not doing it properly. So the disappointment could be a very harmful situation.

Some interviewees explained that the problem of underrepresentation of patients in health care companies’ decision-making processes in research and innovation has become more pronounced since the economic crisis. They seemed to suggest that involving patients in research could improve the quality of the health care product. However,

because of the economic pressure, and the challenge of introducing innovation, I think, my concern is that a lot of players are not paying enough attention to the patient, . . . what the patient needs. So that could be in safety, that could be a compromising on the service. So I think a lot of the economic pressure could compromise what the patient will experience at the end.

### Competitiveness

Another prominent reason for not involving different stakeholders, particularly patients, in research in health care is the companies’ concerns about having less competitive products. Ten of the interviewees (56%) underlined the challenge of balancing the need to achieve financial profit with the need to find the resources to conduct activities in a responsible manner. They reported that a major problem is the fact that not all companies apply RRI. The companies that adopt RRI may end up being less competitive, because they will be more expensive compared to companies that are not adopting RRI. When asked about the main concerns regarding the adoption of RRI, one interviewee responded:

If competitors don’t follow responsible research and innovation, but we in the company, yes, it could become our disadvantage.

Some interviewees mentioned that the additional cost of adopting RRI will particularly affect SMEs. For example, when an interviewee was asked about stricter regulations for making RRI compulsory for all companies, the response was that this may place small companies in a disadvantaged position compared to big companies:

To some extent it (imposing stricter regulations for implementation of RRI) limits what we can do, which products we can develop, because the cost for developing medical products will rise incredibly and small companies will not be able to afford it. So I’d say, a discrimination against small companies. This is how I see it. Only the big players in the market, they will be able to follow these rules. Which means you will have enormous costs. And small companies, cannot.

## Discussion

The present study aimed to examine the application of the RRI approach in the health care private sector, especially at SME level. Although there is a general recommendation for inclusive research,^19^ which in health care could be translated as research *with* patients rather than research *on* patients, we have limited evidence of the extent of the actual involvement of patients in research and innovation conducted in medical equipment manufacturers.

The findings show that decisions about research and innovation in the health care industry are made internally in the company with limited involvement from other stakeholders. The stakeholders which are currently involved in research and innovation activities in the private sector (apart from company employees), are funders, experts from either universities or the private sector, medical center administrators, and doctors.

*None of the participants reported involvement of patients in the process of research and innovation in their organization.* Some participants reported that patients are involved only at the final stages of product development, for providing feedback and to report their satisfaction regarding the product. The finding that patients are not involved at all in the early stages of the research process, when research is designed, is troublesome given that there is evidence of a relation between involvement of patients early in the research process and the quality of the health care that is provided.^20-22^

When the key industry representatives were asked to explain why they omit patients from the research process, the most prominent reason given was the additional cost that involvement of patients entails, which will have negative consequences on the competitive power of the product. These findings are in line with Chatfield and Iatridis et al.,^23^ who found that key industry representatives in the ICT sector expressed significant concerns about the adoption of RRI because of the economic consequences of the RRI activities. The economic consequences appear to be of great significance to all participants, but especially to the managers of SMEs, for which even small increases in costs may affect the survival of the company.^24^

The considerable emphasis placed by key industry representatives on the competitiveness of their products shows that it is not only research conducted in the academic sector which has been influenced by the hypercompetitive culture of biomedical science^25^; research conducted in the private sector has also been affected. A key issue for policy makers and researchers to investigate further is therefore to find ways to bring the values of RRI into the foreground, making responsible research and innovation a competitive advantage for both the academic and industry sectors, rather than a barrier.

The other factor that explains the limited involvement of patients in the research and innovation process in the private sector is the beliefs and attitudes of key representatives. Our data show that for the majority of the health care managers, involvement of patients in the process of research is not a priority. The omission of references to patients, even when the interviewees were directly asked about end users, shows that some key representatives in health care do not see the value of involving patients in their research and innovation activities. In some cases high-ranking decision-makers in the manufacturing medical equipment sector considered the involvement of patients in research and innovation activities as problematic; they believe patient involvement could be damaging for the research and innovation process itself, because if the company cannot fulfil their expectations, its reputation will be negatively affected.

On a more positive side, some of the participants showed an appreciation of the value of involving patients in the process of research and innovation, and they acknowledged that the current absence of patients from the process will have negative consequences on the quality of the health care products that are developed. These findings highlight the role of values and moral motivation for the implementation of RRI.^26^ Those participants who seem to value inclusive research reported that they struggle to find a balance between the different and sometimes competing interests of stakeholders involved in research and innovation in health care. One of the greatest challenges reported is to find a balance between private sector funders’ demands for profit and the implementation of RRI, which involves taking patients’ needs and perspectives into consideration, but which is perceived to be expensive.

## Conclusions

The findings of the present study have important implications. The finding that some companies appreciate RRI and expressed the willingness to apply this approach but struggle with balancing economic factors and responsible research suggests that there is a need to provide support to those companies on how to effectively apply RRI. Presentation of successful case studies of the implementation of RRI in companies could be beneficial in supporting SMEs to apply RRI. The finding that some companies do not acknowledge the importance of inclusive research suggests that there is a need to offer more information regarding RRI and its potential benefits for the companies themselves. There is a need for policy-makers to address recommendations for the private sector and find ways to monitor progress in companies regarding inclusive research.

While the present study sheds some light on the implementation of RRI in the private health care sector, further research is required to enrich our understanding of what the private sector considers as obstacles to implementing RRI.

## Notes

1. LuoX, LiH, ZhangJ, ShimJP. Examining multi-dimensional trust and multi-faceted risk in initial acceptance of emerging technologies: An empirical study of mobile banking services. Decision Support Systems 2010 May 31;49(2):222–34.

2. AlbertsB, KirschnerMW, TilghmanS, VarmusH. Rescuing US biomedical research from its systemic flaws. Proceedings of the National Academy of Sciences 2014;111(16):5773–7.10.1073/pnas.1404402111PMC400081324733905

3. See note 2, Alberts et al. 2014:5773–7.

4. LandisSC, AmaraSG, AsadullahK, AustinCP, BlumensteinR, BradleyEW, et al A call for transparent reporting to optimize the predictive value of preclinical research. Nature 2012 Oct 11;490(7419):187–91.2306018810.1038/nature11556PMC3511845

5. OvseikoPV, GreenhalghT, AdamP, GrantJ, Hinrichs-KrapelsS, GrahamKE, et al A global call for action to include gender in research impact assessment. Health Research Policy and Systems 2016 Jul 19;14(1):50.2743205610.1186/s12961-016-0126-zPMC4950803

6. MoherD, GlasziouP, ChalmersI, NasserM, BossuytPM, KorevaarDA, et al Increasing value and reducing waste in biomedical research: Who’s listening? The Lancet 2016 Apr 15;387(10027):1573–86.10.1016/S0140-6736(15)00307-426423180

7. IoannidisJP, GreenlandS, HlatkyMA, KhouryMJ, MacleodMR, MoherD, et al Increasing value and reducing waste in research design, conduct, and analysis. The Lancet. 2014 Jan 17;383(9912):166–75.10.1016/S0140-6736(13)62227-8PMC469793924411645

8. Statistical Office of the European Communities. *EUROSTAT: Regional statistics: Reference Guide*; 2017; available at http://ec.europa.eu/eurostat/statistics-explained/index.php/Archive:Europe_2020_indicators_-_research_and_development (last accessed 1 Feb 2017).

9. European Commission. *Responsible Research & Innovation*; available at https://ec.europa.eu/programmes/horizon2020/en/h2020-section/responsible-research-innovation (last accessed 29 Jul 2018).

10. IordanouK. From theory of mind to epistemic cognition: A lifespan perspective. Frontline Learning Research 2016a;4(5):106–19.

12. IordanouK, KendeouP, BekerK. Argumentative reasoning In: SandovalW, GreeneJ, BråtenI, eds. Handbook of Epistemic Cognition. New York: Routledge; 2016:39–53.

13. ChatfieldK, BorsellaE, MantovaniE, PorcariA, StahlBC. An investigation into risk perception in the ICT industry as a core component of responsible research and innovation. Sustainability. 2017b Aug 11;9(8):1424.

14. World Health Organization. The World Health Report—Primary Health Care (Now More Than Ever). Geneva, Switzerland: World Health Organization Press; 2008.

15. ClancyC, CollinsFS. Patient-Centered Outcomes Research Institute: The intersection of science and health care. Science Translational Medicine 2010 Jun 23;2(37):37cm18.10.1126/scitranslmed.300123520574065

17. KayeJ, CurrenL, AndersonN, EdwardsK, FullertonSM, KanellopoulouN, et al From patients to partners: Participant-centric initiatives in biomedical research. Nature Reviews Genetics 2012 May 1;13(5):371–6.10.1038/nrg3218PMC380649722473380

18. ChatfieldK, IatridisK, StahlBC, PaspallisN. Innovating responsibly in ICT for ageing: Drivers, obstacles and implementation. Sustainability 2017b Jun 7;9(6):971.

19. See note 9, European Commission 2014 Jun 28.

20. See note 15, Clancy, Collins 2010:37cm18.

22. See note 17, Kaye et al. 2012:371–6.

23. See note 18, Chatfield et al. 2017b:971.

24. Schroeder D. *D1.2 Case Study Descriptions. Deliverable of the Horizon 2020 Project COMPASS*; 2017; available at https://innovation-compass.eu/wp-content/uploads/2017/07/Deliverable-1_3-Compass-Case-Study-Descriptions.pdf (last accessed 29 April 2017).

25. See note 2, Alberts et al. 2014:5773–7.

26. AuerA, JarmaiK. Implementing responsible research and innovation practices in SMEs: Insights into drivers and barriers from the Austrian medical device sector. Sustainability; 2017 Dec 22;10(1):17.

